# Different Accumulation Profiles of Multiple Components Between Pericarp and Seed of *Alpinia oxyphylla* Capsular Fruit as Determined by UFLC-MS/MS

**DOI:** 10.3390/molecules19044510

**Published:** 2014-04-10

**Authors:** Feng Chen, Hai-Long Li, Yin-Feng Tan, Wei-Wei Guan, Jun-Qing Zhang, Yong-Hui Li, Yuan-Sheng Zhao, Zhen-Miao Qin

**Affiliations:** 1 School of Pharmacy, Hainan Medical University, Haikou 571101, China; 2 Hainan Provincial Key Laboratory of R&D of Tropical Herbs, Hainan Medical University, Haikou 571101, China; 3 Haikou Municipal Key Laboratory of R&D of Li Nationality Herbs, Hainan Medical University, Haikou 571101, China; 4 The Hamner Institutes for Health Sciences, Research Triangle Park, NC 27709, USA

**Keywords:** secondary metabolites, *A. oxyphylla* capsular fruits, diarylheptanoids, flavonoids, nootkatone, production region, UFLC-MS/MS

## Abstract

Plant secondary metabolites are known to not only play a key role in the adaptation of plants to their environment, but also represent an important source of active pharmaceuticals. *Alpinia oxyphylla* capsular fruits, made up of seeds and pericarps, are commonly used in traditional East Asian medicines. In clinical utilization of these capsular fruits, inconsistent processing approaches (*i.e.*, hulling pericarps or not) are employed, with the potential of leading to differential pharmacological effects. Therefore, an important question arises whether the content levels of pharmacologically active chemicals between the seeds and pericarps of *A. oxyphylla* are comparable. Nine secondary metabolites present in *A. oxyphylla* capsular fruits, including flavonoids (e.g., tectochrysin, izalpinin, chrysin, apigenin-4',7-dimethylether and kaempferide), diarylheptanoids (e.g., yakuchinone A and B and oxyphyllacinol) and sesquiterpenes (e.g., nootkatone), were regarded as representative constituents with putative pharmacological activities. This work aimed to investigate the abundance of the nine constituents in the seeds and pericarps of *A. oxyphylla*. Thirteen batches of *A. oxyphylla* capsular fruits were gathered from different production regions. Accordingly, an ultra-fast high performance liquid chromatography/quadrupole tandem mass spectrometry (UFLC-MS/MS) method was developed and validated. We found that: (1) the nine secondary metabolites were differentially concentrated in seeds and fruit capsules; (2) nootkatone is predominantly distributed in the seeds; in contrast, the flavonoids and diarylheptanoids are mainly deposited in the capsules; and (3) the content levels of the nine secondary metabolites occurring in the capsules varied greatly among different production regions, although the nootkatone levels in the seeds were comparable among production regions. These results are helpful to evaluating and elucidating pharmacological activities of *A. oxyphylla* capsular fruits. Additionally, it may be of interest to elucidate the mechanisms involved in the distinct accumulation profiles of these secondary metabolites between seeds and pericarps.

## 1. Introduction

Plants have colonized the vast majority of the terrestrial surface on the Earth and largely contributed to the terrestrial biomass by volume and weight. Meanwhile, plants have evolved biochemical pathways that allow them to synthesize a wealth of chemicals, *i.e.*, secondary metabolites that increase plants’ overall ability to survive and overcome local challenges [1]. For instance, these metabolites act, at least in part, as protectants (often described as being antibiotic, antifungal and antiviral agents) for plant bodies against herbivores and pathogens, as well as from physical stresses like ultraviolet light and heat [2]. In general, secondary metabolites are end-products of relatively lengthy biosynthetic pathways. After accumulation under certain conditions, these products are then transported within the plant, or organ, to a site of storage (e.g., to the cell vacuole) or are excreted and deposited on the surface [3]. Furthermore, variations exist in the distribution of secondary metabolites among different plant parts. It appears that these products are often concentrated in the most vulnerable tissues.

In light of their biological activities, plant secondary metabolites have been used for centuries in traditional medicine and as valuable compounds for pharmaceutical industries nowadays. Surveys have shown that a quarter of the drug molecules used in Western countries is of natural plant origin [4]. *A. oxyphylla* (Zingiberaceae) is an herbaceous perennial plant and its capsular fruits are commonly used in traditional East Asian medicine for the treatment of diarrhea [5], intestinal disorders [6], dieresis [7], frequent urination and loss of bladder control [8]. Modern pharmacological studies have demonstrated that *A. oxyphylla* capsular fruits have anti-inflammatory activities [9,10], anti-allergy [11], anti-ulcer [12] and neuroprotective roles [13,14]. 

The secondary metabolites of *A. oxyphylla* capsular fruits include flavonoids (e.g., tectochrysin, izalpinin, chrysin, apigenin-4',7-dimethylether and kaempferide), diarylheptanoids (e.g., yakuchinone A, yakuchinone B and oxyphyllacinol), sesquiterpenes (e.g., nootkatone), volatile oils, steroids and their glycosides, *etc.* [15,16]. Recently, we reported the content levels of nine representative compounds occurring in the capsular fruits of *A. oxyphylla* harvested at different times [17]. Meanwhile, we measured the nine important phytochemicals in rhizomes and leaves of *A. oxyphylla* and found that the concentrations of these compounds were comparable between rhizomes and leaves, but both were significantly less than in fruits (this data will be published elsewhere). Therefore, the nine monitored secondary metabolites are accumulated preferentially in fruits, instead of rhizomes or leaves.

**Figure 1:**
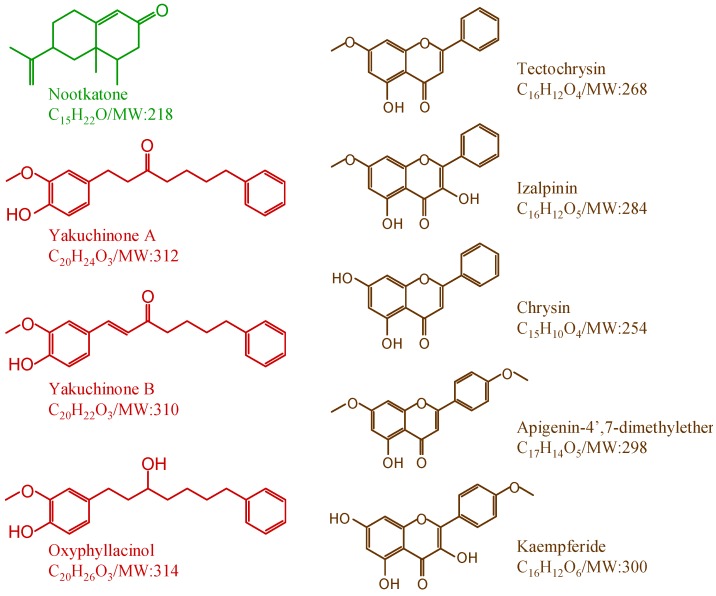
Chemical structures of nine secondary metabolites occurring in *A. oxyphylla* capsular fruits.

As well known, the majority of herbal drugs used in Traditional Chinese Medicine appear to be subjected to some form of pretreatment (processing). During processing*,* the crude herbs are processed into smaller pieces through procedures such as cleaning, cutting, stir baking, so as to obtain processed herbs fulfilling the requirements of therapy, dispensation and making preparations, thus assuring the safety and efficacy of the drugs [18]. For *A. oxyphylla* capsular fruits, there are different processing approaches. As a result, some clinicians make use of the whole capsular fruit; however, others only utilize its seed after hulling the capsule from capsular fruit [19,20]. In our lab, we have found that ethanol extract of seed and capsule from *A. oxyphylla* demonstrated different anti-diarrheal activities. Hence, it seems that the abundance of the secondary metabolites in the seeds may be different from those in the capsules of *A. oxyphylla*. In order to testify this hypothesis, we gathered 13 different batches of *A. oxyphylla* capsular fruits from Hainan Province and Guangdong Province in China; nine representative secondary metabolites (Figure 1) occurring in seed or capsule of *A. oxyphylla* were measured using ultra fast high performance liquid chromatography/quadrupole tandem mass spectrometry (UFLC-MS/MS). Notably, we found that nootkatone was predominantly distributed in the seeds, while the flavonoids and diarylheptanoids were almost exclusively deposited in the capsules. The information gained is helpful to evaluating and elucidating the pharmacological activities of *A. oxyphylla* capsular fruits. 

## 2. Results and Discussion

### 2.1. Selection of the Extraction Method

According to the recently published papers [8,17] by our research team, we chose 70% ethanol (*v*/*v*) as the extract solvent and ultrasonic extraction (40 KHz, 80 W, Kunshan Ultrasonic Instruments, Kunshan, China) was used as the extraction method in this study. The number of repetitions of extraction (30 min every time) was evaluated. The results showed that the recoveries of the nine secondary metabolites were about 90% by single extraction but almost 100% through triple extraction for seed and pericarp samples. Therefore, the sample was macerated with 25 mL of 70% ethanol and then ultrasonicated three times for 30 min each time. 

### 2.2. Analytical Method Validation

**Figure 2:**
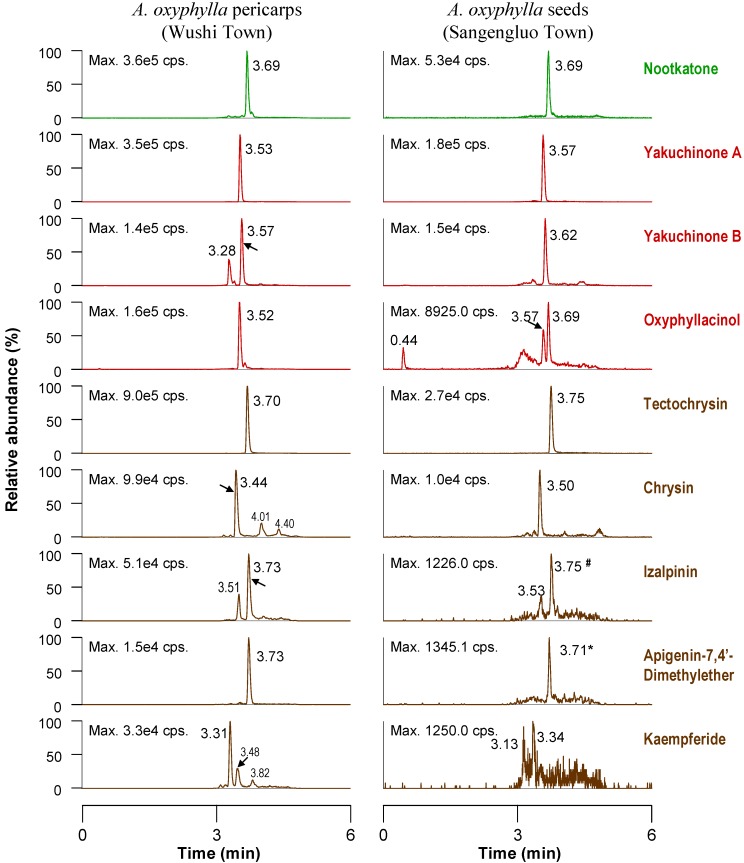
Extracted ion chromatograms of the nine representative constituents from *A. oxyphylla* pericarps and seeds. #: This chromatogram comes from the seeds’ sample collected from Maoyang town; *: This chromatogram comes from the seeds’ sample collected from Yangjiang city.

In order to enhance the analytical efficiency [8,17], we evaluated two shorter chromatographic columns, *i.e.*, Phenomenex Synergi Fusion-RP column (4 μm, 2.00 mm i.d. × 50 mm) and the Phenomenex Luna C_18_ column (5 μm, 2.0 mm i.d. × 50 mm). The results showed the former one provided chromatograms with better peak resolution within a shorter time. All the monitored constituents were mainly eluted within 3.2–3.8 min during a 6-min gradient program (Figure 2). The proposed UFLC-MS/MS method for quantitative analysis was validated by assessing the linearity, LOD, LOQ, intra-day and inter-day precisions and accuracy. All calibration curves showed good linearity (r > 0.9937) within the tested concentration ranges, and the overall LODs and LOQs were in the range of 0.10–5.83 ng/mL and 1.0–11.7 ng/mL, respectively. As shown in Table 1, the RSD values of intra- and inter-day variations of the nine secondary metabolites occurring in seeds were almost less than 10%. The overall recoveries fell between 93.0% and 107% with RSD less than 6% and 10% for seeds and pericarps, respectively. These results indicated that the established method was accurate and reliable. 

### 2.3. Quantitative Analysis of Seed and Pericarp Samples

**Figure 3:**
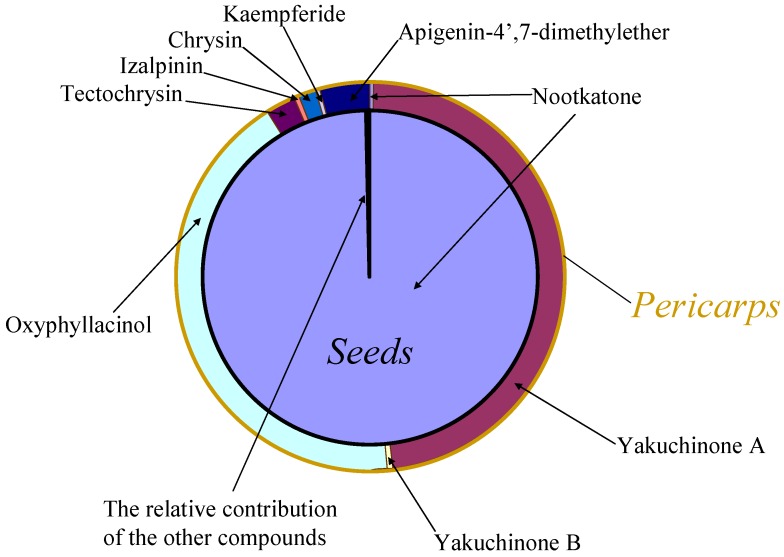
Relative average content levels of nine compounds occurring in seeds and pericarps of *A. oxyphylla*.

Our UFLC-MS/MS method was then applied to detect the nine natural products present in seeds and pericarps of *A. oxyphylla*. All nine compounds were identified by comparing the *m/z* ion pairs and retention times with those of reference standards. The quantitative analysis results are shown in Table 2 and Table 3 for the pericarps and seeds, respectively. In the *A. oxyphylla* seeds (Table 3), the dominant chemical was nootkatone (Figure 3) and its average content from different regions was 2.8 mg/g (0.28%). The diarylheptanoids and flavonoids were distributed in the seeds at very low levels (almost undetectable), especially the flavonoids (Table 3). On the contrary, in pericarps nootkatone (Table 2) was a minor constituent (Figure 3) at a mean concentration of 0.04 mg/g (0.004%), which was only ~1/70 of that in seeds. In addition, the levels of nootkatone in seeds from different production regions were comparable, with a RSD value of 15.3%. 

**Table 1:** The intra-day precision, inter-day precision and recovery test for the current LC-MS/MS method.

Compound	Nootkatone	Yakuchinone A	Yakuchinone B	Oxyphyllacinol	Tectochrysin	Izalpinin	Chrysin	Kaempferide	Apigenin-7,4'-dimethylether
*Pericarps of A. oxyphylla*									
Precision (RSD, %)									
Intraday (n = 6)	1.63	6.59	1.23	6.00	2.31	4.64	4.31	7.02	2.75
Interday (n = 15)	2.74	10.7	1.81	4.18	2.97	5.28	8.11	7.99	3.39
Recovery									
Mean ± SD, %, n = 6	105.8 ± 3.1	100.0 ± 9.9	105.4 ± 5.3	103.2 ± 4.2	107.6 ± 3.8	93.34 ± 5.88	102.0 ± 8.8	96.91 ± 3.26	97.13 ± 6.12
RSD (%)	2.93	9.90	5.03	4.07	3.53	6.30	8.63	3.36	6.30
*Seeds A. oxyphylla*									
Precision (RSD, %)									
Intraday (n = 6)	4.08	2.62	—— ^a^	3.48	1.20	——	4.67	——	——
Interday (n = 15)	6.34	4.40	——	6.74	5.47	——	4.28	——	——
Recovery									
Mean ± SD, %, n = 6	95.67 ± 5.57	101.8 ± 4.23	——	98.40 ± 3.59	106.7 ± 3.05	——	96.48 ± 5.56	——	——
RSD (%)	5.82	4.16	——	3.65	2.86	——	5.76	——	——

^a^ Below the LOQ.

**Table 2:** Content levels (μg/g, mean ± SD) in pericarps of *A. oxyphylla* collected from different production regions.

Production regions	Nootkatone	Yakuchinone A	Yakuchinone B	Oxyphyllacinol	Tectochrysin	Izalpinin	Chrysin	Kaempferide	Apigenin-7,4'-dimethylether
Pericarps (**1**)	46.8 ± 4.2	2961 ± 164	21.1 ± 0.8	2579 ± 106	201 ± 9	47.9 ± 1.6	60.9 ± 1.9	20.1 ± 0.9	268 ± 15
Pericarps (**2**)	35.1 ± 3.4	6440 ± 282	63.6 ± 7.6	5519 ± 177	139 ± 17	32.5 ± 2.3	165 ± 18	27.6 ± 2.5	242 ± 35
Pericarps (**3**)	58.5 ± 3.9	2913 ± 149	23.3 ± 0.7	2552 ± 228	278 ± 11	64.5 ± 3.2	69.0 ± 3.8	25.6 ± 0.6	186 ± 7
Pericarps (**4**)	48.9 ± 6.2	4146 ± 270	35.0 ± 3.6	3666 ± 342	170 ± 24	42.0 ± 4.2	145 ± 8	38.9 ± 2.9	304 ± 29
Pericarps (**5**)	158 ± 17	5390 ± 752	45.3 ± 6.3	4647 ± 514	266 ± 5	59.7 ± 3.4	245 ± 21	36.2 ± 2.7	308 ± 6
Pericarps (**6**)	15.8 ± 1.0	1772 ± 62	90.5 ± 5.8	1699 ± 113	187 ± 9	30.7 ± 2.0	64.2 ± 2.7	23.9 ± 1.5	519 ± 28
Pericarps (**7**)	13.0 ± 0.4	3473 ± 449	91.5 ± 91.5	3177 ± 308	208 ± 15	33.5 ± 1.9	109 ± 7	12.4 ± 0.8	232 ± 23
Pericarps (**8**)	6.56 ± 0.04	2799 ± 339	110 ± 3	2724 ± 287	305 ± 14	38.3 ± 0.9	76.1 ± 5.6	14.9 ± 1.0	796 ± 14
Pericarps (**9**)	48.9 ± 6.2	3853 ± 345	44.9 ± 3.0	3641 ± 118	180 ± 12	38.6 ± 2.2	137 ± 8	30.1 ± 1.5	325 ± 23
Pericarps (**10**)	6.28 ± 0.22	5916 ± 600	66.4 ± 4.0	5230 ± 232	138 ± 10	31.1 ± 1.7	101 ± 11	23.2 ± 2.5	298 ± 20
Pericarps (**11**)	25.5 ± 1.9	1114 ± 240	75.0 ± 5.3	1114 ± 80	192 ± 8	21.4 ± 1.5	98.8 ± 3.3	19.8 ± 0.4	378 ± 17
Pericarps (**12**)	15.2 ± 1.2	6657 ± 504	16.4 ± 0.5	5656 ± 485	213 ± 4	66.7 ± 2.5	212 ± 8	18.4 ± 0.1	121 ± 5
Pericarps (**13**)	59.8 ± 2.9	3073 ± 189	98.5 ± 3.4	2973 ± 112	186 ± 2	30.2 ± 1.6	98.6 ± 5	20.0 ± 0.4	441 ± 20

**Table 3:** Content levels (μg/g, mean ± SD) in seeds of *A. oxyphylla* collected from different production regions.

Production regions	Nootkatone	Yakuchinone A	Yakuchinone B	Oxyphyllacinol	Tectochrysin	Izalpinin	Chrysin	Kaempferide	Apigenin-7,4'-dimethylether
Seeds (**1**)	2416 ± 134	2.74 ± 0.14	——	7.77 ± 0.13	0.77 ± 0.05	——	0.55 ± 0.04	——	——
Seeds (**2**)	3028 ± 77	0.90 ± 0.03	——	6.64 ± 0.09	0.41 ± 0.03	——	0.59 ± 0.02	——	——
Seeds (**3**)	2888 ± 217	1.13 ± 0.03	——	5.05 ± 0.38	0.44 ± 0.02	——	0.53 ± 0.04	——	——
Seeds (**4**)	3098 ± 127	1.11 ± 0.00	——	5.36 ± 0.26	——	——	0.64 ± 0.01	——	——
Seeds (**5**)	2945 ± 269	1.24 ± 0.07	——	6.56 ± 0.26	0.65 ± 0.01	——	1.80 ± 0.09	——	——
Seeds (**6**)	3692 ± 197	—— ^a^	——	5.63 ± 0.12	——	——	——	——	——
Seeds (**7**)	2878 ± 171	2.94 ± 0.02	——	5.63 ± 0.09	0.39 ± 0.02	——	0.76 ± 0.02	——	——
Seeds (**8**)	2522 ± 48	0.40 ± 0.03	——	2.61 ± 0.11	0.40 ± 0.02	——	——	——	1.85 ± 0.09
Seeds (**9**)	2700 ± 112	3.21 ± 0.09	0.46 ± 0.01	9.85 ± 0.30	0.82 ± 0.01	——	1.84 ± 0.05	——	1.47 ± 0.06
Seeds (**10**)	3095 ± 31	0.43 ± 0.05	——	5.91 ± 0.22	——	——	0.43 ± 0.01	——	——
Seeds (**11**)	2750 ± 36	1.11 ± 0.00	——	4.22 ± 0.11	0.61 ± 0.05	——	0.43 ± 0.02	——	1.00 ± 0.04
Seeds (**12**)	1898 ± 117	3.90 ± 0.05	——	8.54 ± 0.27	——	——	0.60 ± 0.05	——	——
Seeds (**13**)	2501 ± 105	5.17 ± 0.10	——	10.7 ± 0.22	1.08 ± 0.03	——	1.39 ± 0.03	——	2.03 ± 0.10

^a^ Below the LOQ.

Why was nootkatone almost uniformly accumulated in the *A. oxyphylla* seeds? This secondary constituent might be produced to benefit the plant. As a sesquiterpene compound, nootkatone was found to possess insecticidal activity against larvae of *Drosophila melanogaster* Meigen [21], nymphal *Ixodes scapularis* Say [22,23], *Xenopsylla cheopis* (Rothchild) and *Aedes aegypti* (L.) adults [23]. Nootkatone was also a strong repellent and toxicant to Formosan subterranean termites [24,25,26]. As we know, many secondary metabolites are toxic in varying degrees to other forms of life, if not to mammals, then to insects and mollusks or to pathogenic bacteria and fungi [3]. Seeds are important organs for the reproduction and spreading of flowering plants. Therefore, the nootkatone occurring in *A. oxyphylla* seeds might be toxic to insects and herbivores and could be a useful defense agent. 

Like the five flavonoid constituents, all three of the diarylheptanoids were more abundant in the *A. oxyphylla* capsules, rather than seeds, although their concentrations were greatly different (Table 2). Yakuchinone A and oxyphyllacinol were the major constituents, and their levels were comparable (Figure 3). The average contents for different production regions were 3.9 mg/g (0.39%) and 3.5 mg/g (0.35%), respectively. The accumulation of yakuchinone B in the capsules was significantly less than the other two diarylheptanoids, with an average content of 0.06 mg/g (0.006%; *i.e.*, ~1/60). The rank order of the five flavonoids concentrated in the fruit capsules by content levels was as follows: apigenin-4',7-dimethylether (~0.3 mg/g) > tectochrysin (~0.2 mg/g) > chrysin (~0.1 mg/g) > izalpinin (~0.04 mg/g) > kaempferide (~0.02 mg/g). Meanwhile, as for the same constituent, the content levels of these natural products varied greatly across production regions (Table 2), which may be explained, at least in part, by their different habitat environments and harvest times.

The mechanisms involved in this selective distribution of the diarylheptanoids and flavonoids in the capsules instead of seeds have not yet been clarified. A major ecological role of flavonoids may be related to their important anti-fungal and anti-bacterial activities. A number of such flavonoids have been identified in the surface waxes of leaves or fruits at concentrations sufficient to prevent germination of hostile fungal spores [3]. Some other activities such as anti-feedant properties and feeding barrier, *etc.* might be the ecological roles for these flavonoids. Furthermore, flavonoids as antioxidants in plants may effectively control key steps of cell growth and differentiation, thus regulating the development of the whole plant and individual organs [27]. Pharmacological studies have shown that yakuchinone A and B exhibit anti-oxidative, anti-inflammatory, anti-tumor and anti-bacterial activities [10,28], but the physiological roles of these diarylheptanoids in *A. oxyphylla* itself remain largely unknown. 

## 3. Experimental

### 3.1. Chemicals and Materials

Reference standard of nootkatone (purity, 98%; similarly hereinafter) was purchased from Sigma-Aldrich (St Louis, MO, USA). Yakuchinone A (98%), yakuchinone B (98%) and oxyphyllacinol (98%) were purchased from Chenfun Medical Technology (Shanghai) Co., Ltd. (Shanghai, China). Tectochrysin, izalpinin, chrysin, kaempferide and apigenin-4',7-dimethylether were separated and identified from *A. oxyphylla* by Prof. Zhang (Hainan Provincial Key Laboratory of R&D on Tropical Medicinal Plants, Haikou, China). On the basis of UV, NMR and MS analysis, the structures of isolated reference standards were confirmed and their purities were 98.0% or greater as determined by HPLC-PDA-MS. HPLC-grade methanol and acetonitrile were from Merck (Darmstadt, Germany). HPLC-grade formic acid was purchased from Aladdin Industrial Inc. (Shanghai, China). HPLC-grade water was prepared by double-distillation of de-ionized water. The other chemical reagents of analytical grade or better were obtained from Hainan YiGao Instrument Co., Ltd (Haikou, China). The chemical structures of the nine secondary metabolites are shown in Figure 1.

**Figure 4:**
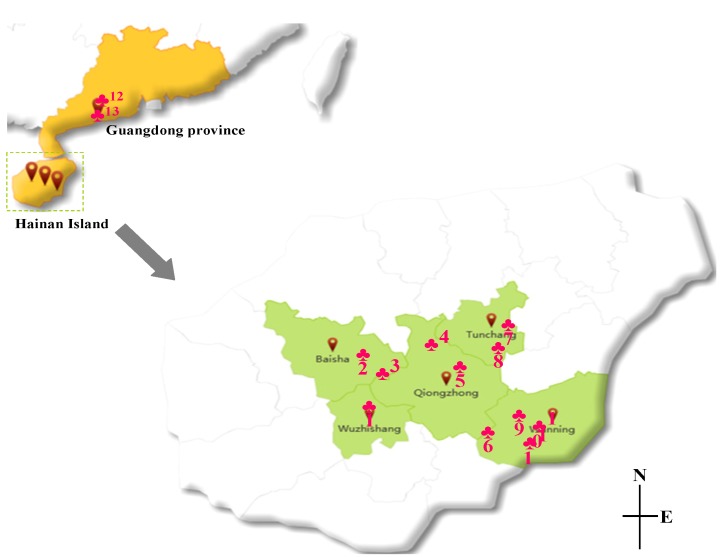
The relative locations of thirteen production regions of *A. oxyphylla* capsular fruits. These production regions are given ID numbers in parentheses. Maoyang Town, Wuzhishan City (**1**); Yacha Town, Baisha Li Autonomous County (**2**); Yinggeling Natural Reserve, Baisha Li Autonomous County (**3**); Limushan Town, Qiongzhong Li Autonomous County (**4**); Wushi Town, Qiongzhong Li Autonomous County (**5**); Changxing village, Heping town, Qiongzhong Li Autonomous County (**6**); Xinxing Town, Tunchang County (**7**); Wupo Town, Tunchang County (**8**); Sangengluo Town, Wanning City (**9**); Changfeng Town, Wanning City (**10**); Xinglong, Wanning City (**11**); Daba Town, Yangdong County (**12**); and Jiangcheng district, Yangjiang City (**13**). ID numbers from **1** to **11** are from Hainan Province; **12** and **13** are from Guangdong Province, China.

The *A. oxyphylla* capsular fruits from different regions are as follows: Maoyang Town, Wuzhishan City (**1**); Yacha Town, Baisha Li Autonomous County (**2**); Yinggeling Natural Reserve, Baisha Li Autonomous County (**3**); Limushan Town, Qiongzhong Li Autonomous County (**4**); Wushi Town, Qiongzhong Li Autonomous County (**5**); Changxing village, Heping town, Qiongzhong Li Autonomous County (**6**); Xinxing Town, Tunchang County (**7**); Wupo Town, Tunchang County (**8**); Sangengluo Town, Wanning City (**9**); Changfeng Town, Wanning City (**10**); Xinglong, Wanning City (**11**); Daba Town, Yangdong County (**12**); and Jiangcheng district, Yangjiang City (**13**). The production regions are given ID numbers in parentheses. ID numbers from **1** to **11** are from Hainan Province; **12** and **13** are from Guangdong Province, China. The locations of these production regions are depicted in the map in Figure 4. These samples were identified by Prof. Jian-ping Tian at Hainan Medical University. The specimens were deposited in the Hainan Provincial Key Laboratory of R&D of Tropical Herbs, Haikou 571101, Hainan province, China. 

### 3.2. Preparation of Sample Solutions

All the *A. oxyphylla* capsular fruits were dried in an electric thermostatic drying oven (DHG-9240A, Yiheng Scientific Instruments, Shanghai, China) at 40 °C overnight. The freshly dried capsular fruits were manually shucked and separated into seeds and fruit capsules, which were smashed using a high-speed smashing machine (FW100, Taisite Instrument, Tianjin, China) and then sieved manually through an 80 mesh. The resulting fine powders and residue were mixed evenly. An aliquot (0.5 g) was weighed precisely and macerated with 70% ethanol (25 mL) [17] and then ultrasonicated three times for 30 min each [8]. For each ultrasonication extraction, the resulting extract solutions were centrifuged at 13,000 rpm for 10 min (Kubota 5922, Kubota Corporation, Tokyo, Japan). One mL of supernatant was sampled and the extract solutions left were discarded. The residue was extracted with 70% ethanol two more times. The sampled ethanol extracts (3 mL) were combined, vortexed to homogeneity and centrifuged at 13,000 rpm for 10 min to obtain the supernatant fractions that were frozen at −20 °C until analysis. The extract solutions were diluted with methanol from 10 to 1,000 times before analysis. Finally, a 10 μL aliquot was injected into the LC-MS/MS system for quantitative analysis. 

### 3.3. Analysis of the Nine Secondary Metabolites Occurring in Seeds and Pericarps of A. oxyphylla

The UFLC-MS/MS method has been partly described by Li *et al.* [17] and by Chen *et al.* [29]. Briefly, an AB-SCIEX API 4000 plus mass spectrometer (Toronto, ON, Canada) interfaced via a Turbo V ion source with a Shimadzu Prominence UFLC chromatographic system (Shimadzu Corporation, Kyoto, Japan), which is equipped with two LC-20AD pumps, a model DGU-20A_3R_ degasser unit, a SIL-20A HT auto-sampler and a CTO-20A column oven. The AB-SCIEX Analyst software packages were used to control the UFLC-MS/MS system, as well as for data acquisition and processing. 

Chromatographic separations of prepared samples were achieved using a Phenomenex Synergi Fusion-RP column (2.00 mm i.d. × 50 mm) maintained at 40 °C. The LC mobile phases included H_2_O containing 0.1‰ formic acid as solvent A, and methanol containing 0.1‰ formic acid as solvent B. A specially designed LC binary gradient elution was performed with gradient program as follows: 0% B holding for 1 min; from 0% B to 80% B in 0.01 min; from 80% B to 100% B in 2.5 min; backing to 0% B in 0.01 min and maintaining 2.5 min. 

The mass spectrometer was operated in the electrospray ionization (ESI) positive ion mode with multiple reaction monitoring (MRM) for all the analytes. The pneumatically nebulized ESI spraying was achieved by using inner coaxial nebulizer N_2_ gas of 55 psi through a Turbo V ion Spray probe, a high voltage of +5.0 kV applied to the sprayer tip, and heated dry N_2_ gas of 55 psi at 500 °C from two turbo heaters adjacent to the probe. To prevent solvent droplets from entering and contaminating the ion optics, a curtain N_2_ gas of 45 psi was applied between the curtain plate and the orifice. The collision gas flow was set at level 7. The precursor-to-product ion pairs used for MRM of nootkatone, yakuchinone A and B, oxyphyllacinol, tectochrysin, izalpinin, chrysin, kaempferide and apigenin-4',7-dimethylether were *m/z* 219.2→163.0 (the optimal collision energy, 22 V), 313.2→136.9 (13 V), 311.2→117.0 (30 V), 315.3→137.0 (22 V), 269.1→226.0 (43.5 V), 285.0→242.0 (43 V), 255.1→152.9 (42 V), 301.1→286.0 (37 V) and 299.2→256.0 (45 V) respectively, with a scan time of 20 ms for each ion pair. 

### 3.4. Method Validation

A fully validated method for measurement of the nine compounds from *A. oxyphylla* fruits harvested at different times was described by Li *et al.* [17]. In the current study, the above stated method was modified to separate the nine secondary metabolites occurring in seeds or pericarps of *A. oxyphylla*. Therefore, an additional partial validation was conducted focusing on the method precision and accuracy.

## 4. Conclusions

An UFLC-MS/MS method was developed and validated and successfully applied to quantify the nine major secondary metabolites found in *A. oxyphylla* seeds and pericarps. Quantification results confirmed our hypothesis that the distribution profile of the nine secondary metabolites in the seeds was different from those in the fruit capsules. Nootkatone was predominantly distributed in the seeds, while the flavonoids and diarylheptanoids were detected mainly in the capsules. The contents of the nine secondary metabolites in the capsules were highly variable among the different production regions; however, the nootkatone levels in the seeds were comparable among production regions. The information gained here is helpful for evaluating the pharmacological roles of *A. oxyphylla* capsular fruits. Further work would be required to characterize the mechanisms involved in the selective distribution of these secondary metabolites into the seeds or pericarps. 
